# Transactions Chicago Medical Society

**Published:** 1874-05-15

**Authors:** Will. T. Montgomery


### Society Reports.

### TRANSACTIONS CHICAGO MEDICAL SOCIETY.

*Reported by Will. T. Montgomery.*

REGULAR semi-monthly meeting, May 4th, in the parlor of the Gault House. President Wm. E. Quine in the Chair.

Order for the meeting: Reports of Cases and Exhibition of Pathological Specimens.

The President reported a case of puerperal septicemia, that had occurred in Cook County Hospital during the prevalence of puerperal fever, which seemed to afford striking evidence of the utility of local medication in the disorder.

The patient, a primipara, aged twenty, was delivered after a quick and easy labor—which resulted, however, in bad laceration of the perineum—of a healthy male child, weighing eight and a half pounds. The uterus did not contract very firmly, though an unusual amount of blood was not lost; and the after-pains were not severe. On the second day after delivery, a copious secretion of milk was established, and the lochia was profuse, sanguinolent, and without offensive odor. In the evening, castor oil was given by the nurse without orders from the physician. On the third day the patient seemed bright, vivacious, and cheerful. She had had several movements of the bowels, and complained of nothing but intense pain in the forehead. Her eyes were red, as though she had been weeping; the pupils contracted; tongue coated with a creamy fur; pulse 132, small and soft; respirations 36; temperature 104°; no pain in any part of abdomen, and no gaseous distension; uterus high and flabby; lochia, dark brown, very profuse, and insupportably fœtid; urine, of normal character and quantity, though it had to be drawn off. During the day she experienced a succession of rigors, which were soon followed by increased acceleration of the pulse, elevation of the temperature, and profuse sweating. Two grains of quinine and one grain of opium were directed to be given every three hours; sponge-baths twice a day, and an intra-uterine bath of an aqueous solution of permanganate of potassium three times a day. The last-named procedure was conducted in the following manner: The patient having been placed on a bed-pan, the disinfecting solution, strength one grain to one drachm, to the amount of a pint, was allowed to flow into and out of the uterus through a double canula catheter, from an ordinary nasal douche apparatus. No pain was complained of by the patient from the application. When the first application was made, on the third day after confinement, the pulse was 132; on the fourth day it was 128; on the fifth, 120; on the sixth, 118; on the seventh, 92; on the eighth, 82;—the observations being made at the same time of day. The pulse invariably fell from five to fifteen beats within two hours after the injections, though it quickly rose again, but only once, above the original frequency. On the fifth day, the patient seemed dull and listless; had no pain, and complained only of a feeling of soreness when pressure was made in right iliac region; diarrhœa and profuse sweating; her tongue was coated posteriorly with a creamy fur, while the tip and edges were very red and the papillæ prominent; the abdominal walls flaccid; gurgling in right iliac region; the uterus lower and firmer than before; the lochia abundant still, but much less offensive; urine normal in quantity. The previous prescription was now discontinued, and fifteen drops of turpentine, in emulsion, was directed to be given every four hours alternately with two grains of quinine and four drops of acid. hydrochlor. The patient being unable to retain either of the mixtures, they were omitted, and pills of two grains of quinine, one-sixth grain of morphine, and one-sixtieth grain of strichnine, were given every trhee hours. There was an abatement of all the symptoms corresponding with the decline in the frequency of the pulse, and improvement in character of lochia. On the eighth day the patient felt quite well and strongly desired food, though there was incomplete involution of the uterus. The case presented two points of interest: it was one of pure, uncomplicated septicemia, and was unquestionably influenced more favorably by local than internal medication.

In the discussion of the case, Vice-President Dr. Paoli said he had found the solution of permangan. pot. a very excellent one to use as an injection in offensive uterine discharges. He referred to the fact that uterine injections are condemned by most authors on account of sometimes producing uterine colic. He thought it very difficult to distinguish between puerperal septicemia and puerperal fever.

Dr. Peterson said the amount of force used in giving uterine injections had much to do with producing colic, and that the solution should be passed in very gently.

Dr. Stillians said he had experienced very satisfactory results from the use of uterine injections by means of the double canula, and thought there was no danger of producing uterine colic when it was used.

The President explained that uterine injections had not been given—only uterine baths. He agreed with Dr. Paoli as to the difficulty of distinguishing between the various affections included under the head of puerperal fever. The latter term is used generically: in one instance applied to septicemia, in another to phlebitis, or lymphangitis; in others to cellulitis, metritis, peritonitis, etc. It was not ordinarily difficult to tell when any one or more of these morbid states existed; generally several of them co-existed. The speaker then detailed the points of difference between the various disorders named, which would serve in establishing a diagnosis. In alluding to the etiology of puerperal fever, he said that, while it occurred spontaneously and sporadically, there is a specific and infectious puerperal poison, which is not contagious, however, in the sense that the poison of variola is; the puerperal poison may give rise to one or more of several widely different morbid states, all of which, however, are included under the head of puerperal fever. One patient may contract a metro-peritonitis, or septicemia, or a cellulitis, from another who has neither of the disorders mentioned, but some other form of the disease. A patient may even contract a fatal puerperal fever from one laboring under any of the ordinary infectious diseases.

